# Circadian regulation of locomotion, respiration, and arousability in adult blacklegged ticks (*Ixodes scapularis*)

**DOI:** 10.1038/s41598-024-65498-z

**Published:** 2024-06-26

**Authors:** Jack P. Marshall, Emily Marinko, Amber To, Jilian L. Morejon, Ritika Joshi, Jamien Shea, Allen G. Gibbs, Matthew R. Meiselman

**Affiliations:** 1 School of Life Sciences, University of Nevada-Las Vegas, Las Vegas, NV 89154 USA; 2 Department of Neurobiology and Behavior, Cornell University, Ithaca, NY 14853 USA

**Keywords:** *Ixodes scapularis*, Sleep, Circadian rhythm, Respiration, Arousability, Vector, Circadian rhythms and sleep, Respiration, Behavioural ecology

## Abstract

The blacklegged tick, *Ixodes scapularis*, is an ectoparasitic arachnid and vector for infectious diseases, including Lyme borreliosis. Here, we investigate the diurnal activity and respiration of wild-caught and lab-reared adult ticks with long-term video recording, multi-animal tracking and high-resolution respirometry. We find male and female ticks are in a more active, more arousable state during circadian night. We find respiration is augmented by light, with dark onset triggering more frequent bouts of discontinuous gas exchange and a higher overall volume of CO_2_ respired. Observed inactivity during the day meets the criteria of sleep: homeostatic in nature, rapidly reversible, a characteristic pose, and reduced arousal threshold. Our findings indicate that blacklegged ticks are in a distinct, heightened state of activity and arousability during night and in dark, suggesting this period may carry higher risk for tick bites and subsequent contraction of tick-borne diseases.

## Introduction

Animals utilize many strategies to optimize their behavior, executing energy expenditure not only in the best spatial context, but also in an optimal temporal context. For this, most species rely on a circadian program that dictates the time of day when wakefulness and sleep are appropriate. Predators, for instance, increase their alertness when the chances of encountering prey are highest. The parasitic blacklegged tick, *Ixodes scapularis*, typically finds its blood meals using a strategy known as questing, wherein they perch upon vegetation with legs extended, until it can latch on to a passing host^1^. Their hosts include many organisms known to be either crepuscular or active at night^2,3^. *Ixodes scapularis* appear to be less reliant on light and sight than other arachnids, with a primitive visual system even relative to other hard-bodied ticks^2,4–6^. It is therefore unsurprising that several accounts exist of *I. scapularis* nymphs and adults questing more often in the early morning^7^, late afternoon and night^8–10^ rather than the brightest parts of the day. However, questing ticks are thought to only represent a small portion (~ 6.3%) of the ticks present in an area^11^, and actively questing ticks may or may not be indicative of the population’s circadian rhythm. Moreover, *Ixodid* activity appears to be influenced by a combination of conditions that are heterogenous in the wild, such as region, climate, and satiety^7,12–16^. Questing can be elicited by cues from a potential host, such as kairomones or carbon dioxide^17,18^. It is, therefore, important to know not only when the animals are in the latter stages of questing, but when they are arousable and capable of actively responding to host-associated cues.

We sought to determine the innate circadian activity patterns of wild-caught and lab-reared populations of *I. scapularis*. We designed a custom camera setup for long-term video recording and tracking and examined when and to what degree adult male and female ticks were active. We found sex-specific activity patterns common to the wild and lab-reared populations. In both sexes, activity was highest just after darkness onset. When night was unexpectedly long, some entrained ticks became active at the moment of expected night, suggesting the presence of a surprisingly accurate circadian clock. Further, using a respirometer on individual ticks, we recorded the metabolic rate of individual ticks over a circadian day. Interestingly, during circadian night ticks breathed more frequently, and expelled more carbon dioxide, irrespective of activity levels. We determined that observed activity is homeostatic, and day-associated inactivity is marked by an increased arousal threshold, meeting the criteria for the classical definition of sleep^19^. We posit that ticks are more active and in a heightened stated of alertness during night. Overall, we show that adult *I. scapularis* are most active at night, suggesting greater potential for attachment on hosts that are commonly active after nightfall.

## Results

### A long-term observation overhead camera (LOOC) to measure circadian rhythms

**Figure 1:**
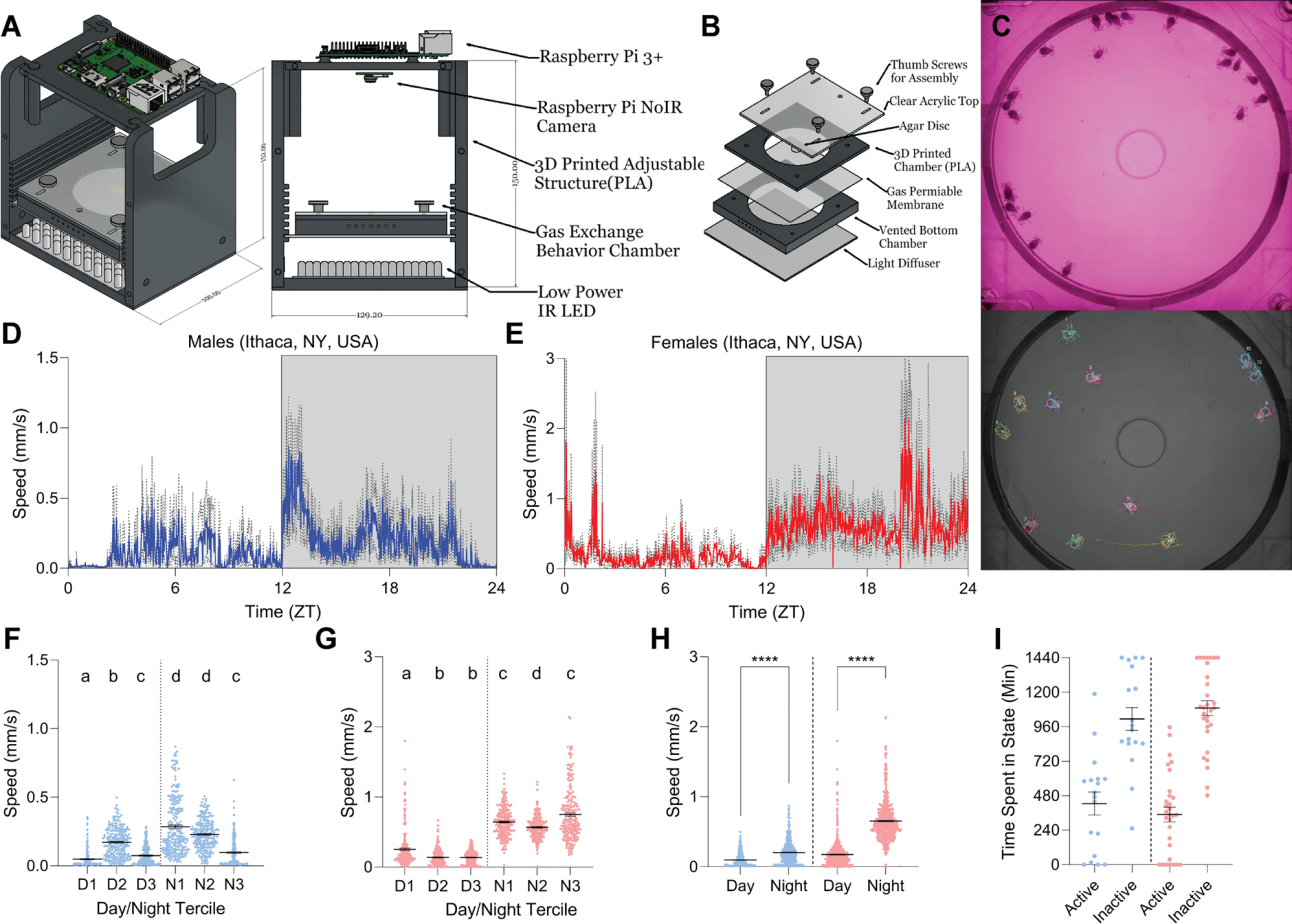
Wild-caught ticks are more active during circadian night. (**A**) Labeled schematic of the Long-term Observation Overhead Camera (LOOC). (**B**) Inserted cylindrical chamber (right) has 65 mm in diameter, 1.5 mm in height arena with ticks and a 1.5% agar disc for humidity within (see methods for details). (**C**) Frame from an example video pre-processing (top) and after T-Rex tracking software (bottom). Ticks are identified as “blobs” and position in video is monitored over time, used to calculate velocity. (**D**–**E**) Average velocity per tick over a 24-h (1440-min) day with 1 min binning (mm/s) for wild-caught (**D**) males (n = 18) and (**E**) females (n = 30). Dark gray dotted line indicates SEM. Gray region of plot delineates night. (**F**–**G**) Average velocity for males (**F**) or females (**G**) from (**D**) and (**E**) at every minute during each 4 h/240 min window of day and night (240 min/points). Letters indicate statistically distinct groups (Mann–Whitney Ranked Sum comparisons, p < 0.05). (**H**) Average velocity for males (left, blue) and females (right, red) from D and E at every minute during each 12 h/720 min window of a circadian day (720 min/points) (unpaired t-test, ****p < 0.0001). (**I**) Time spent moving (active) or at rest (inactive) over a circadian day for each tick tracked in (**D**, left) and (**E**, right). In all panels, error bars indicate mean +/− SEM.

To assess the circadian activity of *I. scapularis* males and females, we designed and built a custom camera system that could fit in an incubator, and record tracking-quality video for multiple consecutive days, LOOC (Long-term Observation Overhead Camera, Fig. 1A–C). We collected wild ticks in Ithaca, New York, USA (42° 27′ 47.4″ N 76° 25′ 37.8″ W) during late morning hours through April and May 2022. Adult *I. scapularis* were collected and divided by sex into groups of 6–12, placed into a vial with a wet kimwipe and placed in an incubator (25 °C, 80% RH, 12:12 L:D) for two days to acclimate, and then inserted into LOOC for circadian assessment. Using T-Rex multi-animal tracking^20^, we tracked average velocity of individual ticks for each minute of a circadian day (Fig. 1D–E). Males and females had distinct activity patterns: Males were more active at midday (ZT4-8), with a peak of activity just after lights off, and tapering activity as night progressed (Fig. 1D,F). Females held consistent activity levels within the day and within the night (Fig. 1G). For both sexes, activity was significantly higher in dark conditions, during the scotophase (Fig. 1H). Males were active 7.09 h/day (± 1.34 h/day S.E.M.) and females active 6.57 h/day (± 0.86 h/day S.E.M.) (Fig. 1I).

**Figure 2:**
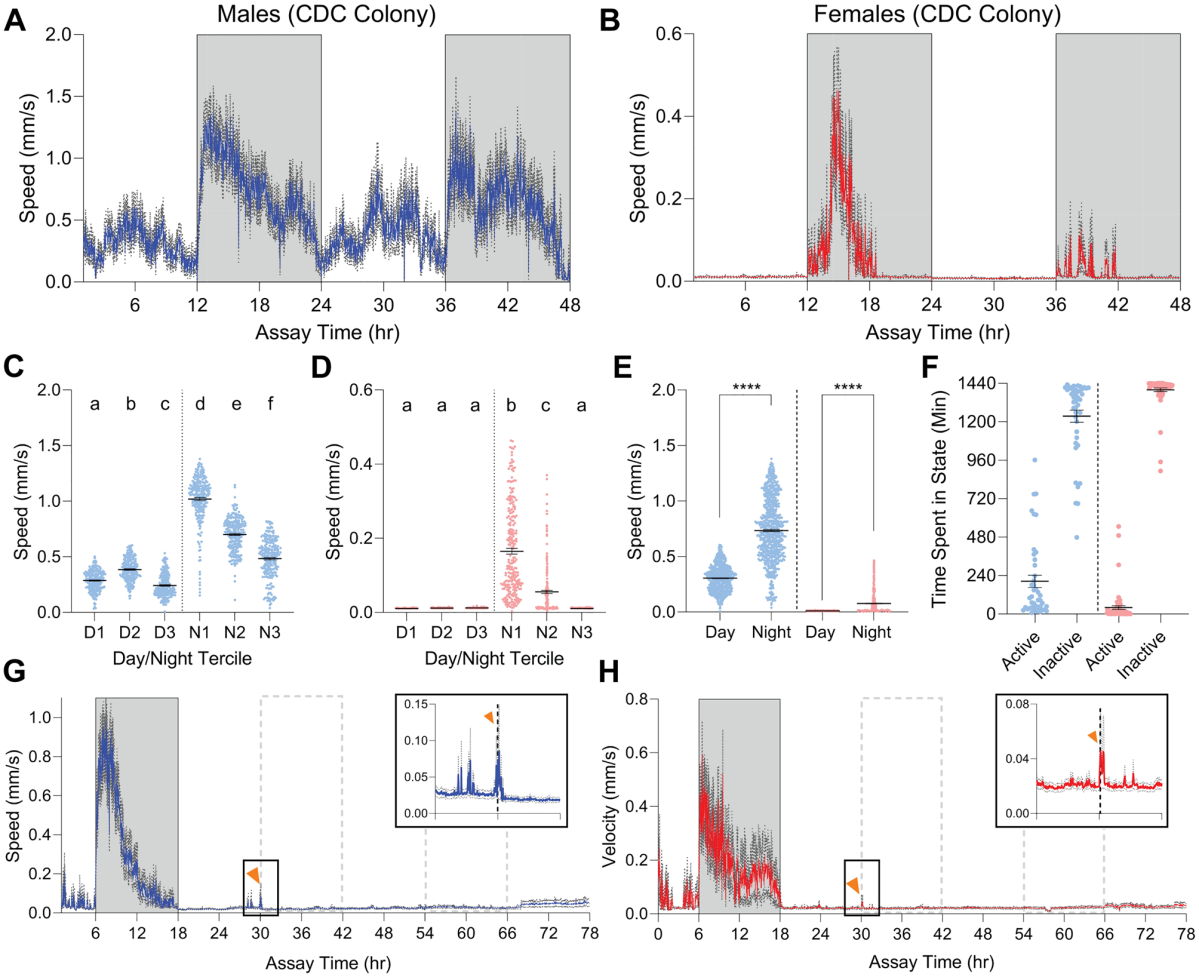
Activity of lab-reared ticks is rhythmic, higher at night. (**A**–**B**) Average velocity per tick over a 48-h (2880-min) window with 1 min binning (mm/s) for lab-reared males (**A**, n = 68) and females (**B**, n = 71). Dark gray dotted line indicates SEM. Gray region of plot delineates night. (**C**–**D**) Average velocity for males (**C**) or females (**D**) from (**A**) and (**B**) at every minute during each 4 h/240 min window of day and night (240 min/points). Letters indicate statistically distinct groups (Mann–Whitney Ranked Sum comparisons, *p* < 0.05). (**E**) Average velocity for males (left, blue) or females (right, red) at every minute during each 12 h/720 min window of first circadian day (720 min/points) (unpaired t-test, ****p < 0.0001). (**F**) Time spent moving (active) or at rest (inactive) over a circadian day for each tick tracked in (**A**, left) and (**B**, right). (**G**–**H**) Average velocity per tick for male (**G**, n = 90) or female (**H**, n = 71) ticks exposed to constant light after first night for 2.5 days (3600 min). Dark gray dotted lines represent SEM. Light gray dotted lines indicate expected dark periods based on prior entrainment. 180 min before and 180 min after initial unexpected light are magnified (black box top right) to emphasize activity bursts (orange arrowheads, dashed line is ZT12). In all panels, error bars indicate mean +/− SEM.

We sought to ensure collection time (mid-morning) and season (late spring), as well as field samples that were of nonhomogenous age, pathogen status, nutritional status, and actively host-seeking, did not bias our data from a representative population. We, therefore, assessed a lab-reared, pathogen-free population from the CDC medical entomology lab^21^ in the same conditions. Surprisingly, while overall activity differed between populations, rhythmicity was similar (Fig. 2A–B). Males showed high activity midday and showed a peak after lights off and tapered during night (Fig. 2C). Females had consistent low activity levels during day, but were active earlier in the night, eventually becoming inactive during the final 4 h (tercile) of the 12-h night (ZT20-24) (Fig. 2D). Both sexes were far more active during the scotophase than photophase (Fig. 2E). Lab-reared males were less active than wild-caught, males only active for 3.41 h/day (± 0.63 h/day S.E.M.), and females active for 0.66 h/day (± 0.19 h/day S.E.M.) (Fig. 2F). Manual scoring confirmed no more than 55% of males and 22% of females were active at any point during the day (Fig. S1A–B).

### Circadian activity pattern requires shifting photophase

It remained unclear if nighttime activity was driven by the absence of light or an innate circadian program. In some organisms, rhythmic activity can persist for days after the loss of environmental stimuli on which to entrain^22–25^. We removed nightly light cycling, exposing both sexes to 66 h of constant light instead of the expected 12 (Fig. 2G–H). Both males and females showed a very similar activity pattern after the first night: a small but consistent burst of activity was observed at 12 h after regime switch (when night should have occurred) and slightly increased activity after 48 h of constant light. Only males reached statistical distinction comparing the 4 h around expected night to preceding day and anteceding night (Fig. S2A–B). These features suggest that nighttime activity is driven in part by an internal clock rather than exclusively by a startle effect or light masking^26^. Interestingly, males held in constant dark showed no evidence of rhythmicity and remained inactive for 48 h after the first night (Fig. S2C). Contrastingly, females held in constant darkness showed a burst of activity roughly 24 h after the first night and again at 48 h (Fig. S2D). Overall, it appears shifting lighting conditions are critical inputs for rhythmic activity.

### *Ixodes scapularis* respiration levels are light-dependent

**Figure 3:**
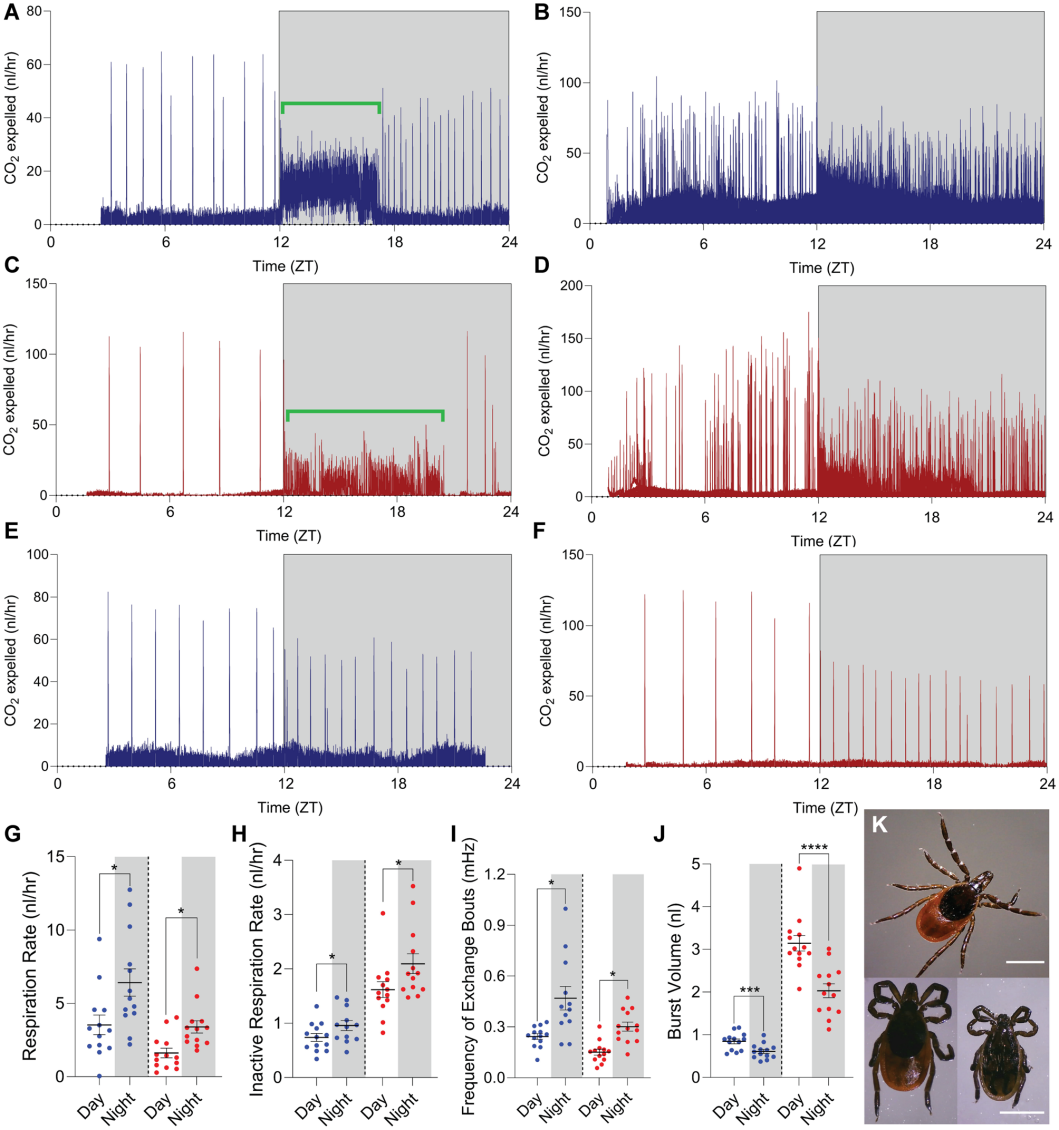
Respiration rate and frequency of gas exchange bouts are light-dependent. (**A**,**C**) Example trace of CO_2_ concentration in flow-through for a single male (**A**) or female (**C**) tick during a circadian day. Gray box indicates night/lights off (ZT12-24), green bracket is an example of movement-associated respiration bouts, where CO_2_ expulsion is sharply elevated. (**B**,**D**) Example traces of CO_2_ concentration in flow-through for 13 male (**B**) or 13 female (**D**) ticks during a circadian day, overlaid. Gray box indicates night/lights off (ZT12-24). (**E**,**F**) Example trace of CO_2_ concentration in flow-through for a single male (**E**) or female (**F**) tick during a circadian day where tick was stationary all day. Gray box indicates night/lights off (ZT12-24). (**G**) Total CO_2_ respiration rate for each male (left) and female (right) tick represented in (**B**,**D**) comparing circadian day (ZT0-12, white) to circadian night (ZT12-24, gray) by sex (n = 13 per sex, pairwise Welch’s t-test, *p < 0.05). (**H**) CO_2_ respiration rate comparing only periods of inactivity for each male (left) and female (right) represented in (B,D) comparing circadian day (ZT0-12, white) and night (ZT12-24, gray)(n = 13 per sex, Mann–Whitney Ranked Sum comparison, *p < 0.05). (**I**) Peak frequency during inactive periods for each male (left) and female (right) tick during represented in (**B**,**D**) comparing circadian day (ZT0-12, white) and night (ZT12-24, gray)(n = 13 per sex, pairwise Welch’s t-test, *p < 0.05). In all panels, error bars indicate mean +/− SEM. (**J**) Average volume of CO2 released during bouts of discontinuous gas exchange during day (white boxes) and night (gray boxes) for males (left, blue) and females (right, red) (n = 13 per sex, pairwise t-test, ***p < 0.001, ****p < 0.0001). (**K**) Example of an active/walking pose (top), compared to sleep pose in female (bottom left) and male (bottom right, white scale bars are 500 µm).

*Ixodes scapularis* are 3-host ticks, and digest their infrequent bloodmeals over periods of months^2^. After preliminary digestion (once ticks are no longer engorged), related hard-bodied ticks enter a state of slowed respiration, marked by periodic opening of spiracles and discontinuous gas exchange^27–29^. Thus, in this inter-meal period, short term meal dynamics have limited influence over metabolic rate, permitting us to attribute short-term changes in respiration dynamics to non-meal factors, such as changing energy demand. We found both sexes of *I. scapularis* respire via discontinuous gas exchange when inactive (Fig. 3A–F). CO_2_ exchange bouts or “bursts” lasted 60–90 s with varying amplitude. As observed in other ticks, movement was associated with a dramatic (5.5-fold for females and 7.2-fold for males) increase in carbon dioxide expelled per minute (Fig. 3A,C, S3K). As determined by tracking, these activity-associated bouts occurred predominantly during circadian night, which contributed to more carbon dioxide expelled overall (Fig. 3G). Surprisingly, the frequency of bouts and overall rate of CO_2_ expulsion was elevated in the dark even when comparing basal respiration in immobile ticks (Fig. 3E–F,H–I). Burst volume was lower at night, suggesting frequency increased to a degree that surpassed the increase in CO_2_ production (Fig. 3J). A difference between basal respiration between night and day was not observed in either the constant light or constant dark regimes (Fig. S3E–J). This suggests that breathing rate and respiration change as a direct response to lighting conditions. Lighting conditions appear to underly metabolic state and could be causally linked to increased activity during night.

### *Ixodes scapularis* sleep during the day

Activity analysis and respirometry showed that wild and lab reared populations enter a circadian pattern of metabolic and behavioral quiescence each day, but it remained unclear whether diurnal inactivity was sleep or a sleep-like state. Sleep typically includes a stereotypical posture, and is defined by three critical criteria: quiescence must be rapidly reversible, be homeostatic in nature, and include a reduced arousal threshold^30^. During bouts of inactivity, we noticed both male and female ticks have a similar posture, notably marked by curling of the prothoracic legs to the basis capitulum, with claw pad tucked just under the palps and pointing forward (Fig. 3K). The second pair of legs is also curled, but in contact with the ground under the prothoracic legs. This posture was always observed during day-associated inactivity, whereas during night ticks had some sedentary periods (speed = 0 mm/s) before and after activity where arms were extended (Fig. S3L).

#### Inactivity is rapidly reversible

**Figure 4:**
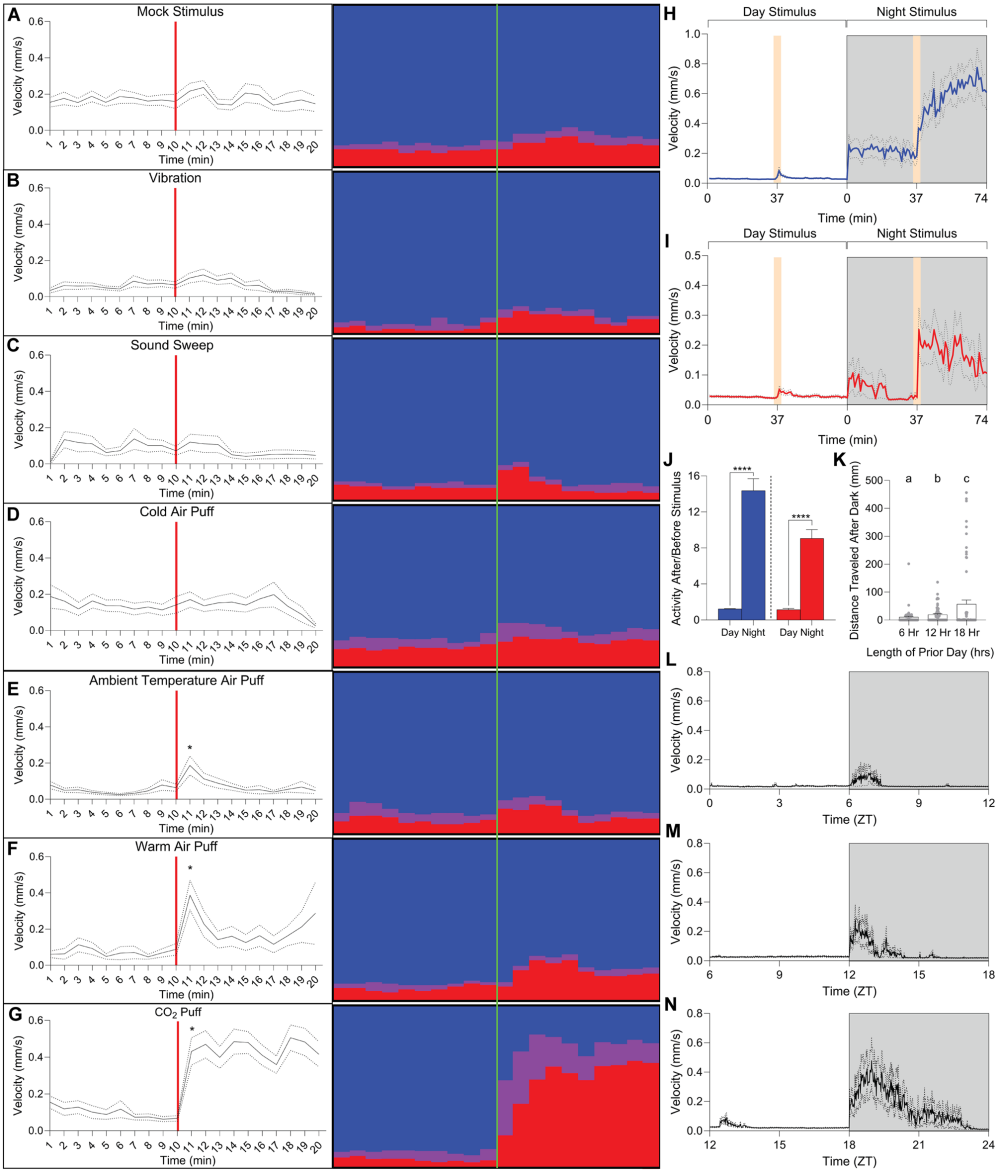
Ticks are more responsive to stimuli at night, nocturnal activity is homeostatic. (**A**–**G**) Tracked activity (left) and ethogram (right, blue is inactivity, purple is stationary waving of prothoracic tarsi, red is position-changing movement) for ticks stimulated with either (**A**) no stimulus, (**B**) vibration, (**C**) sound sweep, (**D**) 4 °C air puff, (**E**) 25 °C air puff, (**F**) 37 °C air puff, or (**G**) a puff of pure carbon dioxide (5 independent trials with 25 female ticks each). Moment of stimulation is marked by red bar (tracking, left) or green line (ethogram, right). Average velocity (mm/s) for group as tracked (left) or manually assessed action score (see methods, right) is presented for 10 min before and after stimulus. Dotted lines indicate SEM, asterisk indicates if latter ten minutes are significantly elevated above first ten (ANOVA, NS, not significant, *p < 0.05). (**H**–**I**) Tracked activity for male (**H**) or female (**I**) ticks 37 min before and after stimulated with a 25 °C air puff at ZT4 (left, white area) or ZT20 (right, gray area). Beige bar indicates moment of stimulus, dotted lines indicate S.E.M. (n = 93, ANOVA, *p < 0.05). (**J**) Ratio of activity in the 37 min after the air puff to the 37 min prior to air puff among active ticks only from H (left, males) and I (right, males). Mann–Whitney Rank Sum comparison, ****p < 0.001, error bars indicate mean +/− SEM. (**K**) Total distance traveled in the 6 h after dark after a day length of indicated time from L-N. a, b, and c represent statistical groups (ANOVA, *p* < 0.05), error bars indicate mean +/− SEM. (**L**–**N**) Tracked activity for female ticks after day length that was either abnormally short (6 h, L, graph starts at ZT0), expected (12 h, M, graph starts at ZT6), or abnormally long (18 h, N, graph starts at ZT12). White space indicates tracking for final 6 h/360 min of day, gray box indicates first 6 h/360 min of dark (n = 68–72), dotted lines indicate S.E.M.

*S*everal hard tick species that have the capacity to enter diapause, a state of dormancy which is not rapidly reversible, and requires specific and persistent cues to terminate^31,32^. To distinguish diurnal inactivity from this or other states where arousability is low, we provided ticks with a battery of acute stimuli that could be easily delivered while ticks were in the LOOC enclosure during a quiescent period (between ZT4 and ZT8). We used both tracking and manual scoring (Fig. 4A–G) of every tick’s posture before and after a stimulus to assess arousal. Ticks did not significantly arouse when stimulated by vibration, sound stimuli, and puffs of 4 °C air (Fig. 4B–D). When puffed with ambient temperature (25 °C) or warm (37 °C) air (Fig. 4E–F), and when puffed with pure carbon dioxide (Fig. 4G), ticks were significantly aroused, first extending and waving their prothoracic legs, and second walking, presumably to seek the source of the stimulus (Example movies S1 and S2). The puff of pure carbon dioxide, contrasting other stimuli, elicited a massive response. Over 90% of ticks transitioned from sleep pose to walking over the minutes following the stimulus-arguing daytime quiescence can be rapidly reversed (Fig. S4A).

#### Activity is homeostatic

Determining whether a homeostasis is maintained by sleep, common practice is to disturb nightly sleep and observe whether sleep rebounds with an increase in bout length after the disruption terminates^30,33–36^. However, our lab-reared population of *I. scapularis* is inactive over 23 h a day, making an expected increase in sleep bout length during sleep rebound after deprivation difficult to observe. We, therefore, decided to manipulate the light cycle, with the expectation that activity levels would correlate with anteceding day length if sleep pressure drove degree of activity. After a 24-h acclimation period, we set lights to turn off after 6 h, 12 h, or 18 h. The total distance traveled was directly proportional to the amount of rest the ticks received during the previous day (Fig. 4K). Notably, activity after 6 h of rest was lower than 12 h, which was significantly lower than activity after 18 h of rest, despite some preceding activity observed at expected dark (~ ZT12), which interrupted rest (Fig. 4L–N).

#### Day time inactivity is coupled with an increased arousal threshold

To be defined as sleep, diurnal inactivity must be distinguished from a quiescent but responsive state. Thanatosis, or death feigning, is an antipredation tactic in which a disturbed insect engages in a pseudo-death pose that has been associated with tick immobility^14^. Contrasting a sleep-like state, thanatosis should not affect arousability. To compare day and night arousability, we selected the stimulus that elicited the minimum significant increase in activity when delivered during day, a puff of ambient temperature air. With this stimulus, we hypothesized margin of increase or decrease in response would be easily visualized. We provided the selected stimulus twice to groups of female and male ticks, once at ZT4 and once at ZT20 with randomized block design and compared response (Fig. 4H–I). Air puffs elicited more activity, which was sustained for a more prolonged period in the context of dark compared to light. The percentage of ticks per group aroused by the same stimulus was also significantly higher at night (Fig. S4B), suggesting that arousal threshold is lower during the night and higher during daytime. Comparing activity in the 37 min before to after stimulus, in male and female ticks, activity increased 23% and 16%, respectively, after stimulus delivered during day, whereas activity increased 1337% and 808%, respectively, to the same stimulus delivered at night (Fig. 4J). As suggested by the respirometry data, this data supports the hypothesis that ticks are in a state of heightened awareness during night. Overall, daytime inactivity appears to meet the definition of sleep.

## Discussion

Activity, respiration and arousal threshold suggest *I. scapularis* males and females are nocturnal animals. We argue that a nightly state of enhanced stimulus responsiveness is an important ecologically-relevant driver of likelihood of host encounters, as time spent questing is limited by risk of desiccation energy waste, and, presumably, predation^37,38^. In the field, *Ixodes* spend a fraction of their day questing^10,39–41^, and our data suggests that this fraction may underrepresent their host-seeking competence. Questing can be driven by host-associated stimuli^17,42,43^, and we show here tick responsiveness to stimuli is heightened during night. Moreover, we show dark is associated with higher respiration rate and more frequent gas exchange even when at rest. Taken together, our findings suggest ticks are in a distinct state during night which likely increases the probability of host encounters.

Wild-type and lab-reared *I. scapularis* appear to have an ingrained circadian rhythm, in all cases most active early in nightfall. While we used an abrupt, less-than-natural oscillation between 250 and 0 lx, several studies of individual populations in natural lighting found similar early-night activity spikes. This regime permitted us to make more obvious the stark contrast in state coordination between light and dark environments. The uncovered commonalities suggest this circadian rhythm is an adaptive and conserved feature. In both cases, we found males showed more relative activity during the day. Sexually dimorphic activity patterns may be related to physiological differences, or adult behavioral differences, such as their role in mating^44^. It is also unclear what role our static photoperiod had in influencing activity, as shifting photoperiod and seasonality influence activity and development^13,31^. Lab-reared male ticks also had more total activity and higher weight-adjusted respiration, which could be the result of their distinct morphology and behaviors. It was interesting to note that males, more active during day, became significantly more active during expected night in the LL regime whereas females barely responded. Females, contrastingly, appeared to cycle activity at night whereas males did not. This may suggest sexually dimorphic elements involved in anticipation, the visual system, or circadian clock.

Respirometry data suggests at least three distinct states; inactive day, inactive night, and active. While unable to compare day versus night activity due to difficulties with peak measurement and paucity of day-associated activity bursts, we observed a clear day vs. night difference in volume of gas exchanged during discontinuous gas exchange bouts and frequency of peaks. The volume and frequency of gas exchanges were light-dependent even comparing inactive ticks, adding a layer of diversity to inactivity. Female ticks were inactive nearly 23 h a day. As respirometry showed a distinction between day and night inactivity, it is unlikely that all 23 h represent a homogenous state. Indeed, a variety of organisms from arthropods to humans have heterogenous stages of sleep with differing roles, contribution to homeostasis, and arousability^45^. The likely implication of such high inactivity levels is that ticks are not always in a reduced-arousal threshold-sleep when inactive. We showed that daytime inactivity met the definition of sleep, and we propose increased respiration during dark is a unique rest state, from which ticks can be rapidly and easily elicited to host-seek or quest by cues from potential hosts. The existence of this third state could explain why ticks remain immobile for multiple circadian days when light cycling is interrupted: not all observed inactivity is sleep.

Sleep and sleep dynamics are critical for vector competence, and detailed information about tick sleep is lacking^46^. We provided sleeping ticks with a variety of stimuli to test their daytime state. We found it surprising ticks were relatively unresponsive to cold air and vibration, as air puffs and vibration interrupt sleep in many other organisms^47–50^. Warm air and CO_2_ are potential indicators of nearby hosts, and it was, thereby, unsurprising both elicited lasting activity which was likely a host/stimulus seeking behavior. While other related ticks actively search for hosts, *I. scapularis* use a sit-and-wait strategy, waiting for hosts to come to them^51^. This calls into question the ultimate purpose of activity observed after CO_2_ stimulus, and a closer approximation of a natural setting will be needed to elucidate. The dramatic difference between day and night response to an ambient-temperature air puff leaves the possibility that several stimuli which did not elicit a response during day, like vibration, may still be important components of host-seeking if presented in the night context.

Finally, the homeostatic nature of activity reinforces the supposition that day inactivity is, indeed, sleep. Compared to inactive periods at night, we see a roughly 23% decline in CO_2_ expelled during day; similar to the 15–30% decline observed during sleep in mammals^52^, and above the 12–15% reduction in CO_2_ expulsion reported in the more closely-related fruit fly^53^. This definition carries connotations suggesting *I. scapularis* are more active and more arousable at night.

Beyond informing risk of tick encounters, this work will allow further investigations of the ecological implications of the activity, respiration, and arousability changes. Further, our classification of this state will allow distinction other quiescent states, such as diapause, from sleep.

## Methods

### Long-term observation overhead camera (LOOC)

Long-Term Observation Overhead Camera (LOOC) was designed and constructed to monitor small organism activity in an enclosed chamber over long periods of time within a small incubator. The 3D-printed chassis made of PLA plastic (Hatchbox black 1.75 mm filament) and is 157 mm in height, 100 mm width, 100 mm length, with 24 parallel circuits of 9 IR LEDs (Everlight Electronics, PN: HIR7393C) in series above an aluminum plate for temperature dispersal. IR is powered by a power supply set to 11.1 V. On top, a Raspberry Pi B + computer is fixed to a height adjustable piece of acrylic, linked to an Arducam 64 MP Raspberry Pi camera module affixed to the underside and covered in a long-pass filter. In middle, chassis has multiple notches for insert. Insert is 3 layers of laser-cut acrylic, a 5 mm thick bottom with a 65 mm depressed well with 4 air ports covered by an air-permeable membrane (MP Biomedicals SKU:097640205), a 1.5 mm thick acrylic spacer with an open circle in the center (65 mm diameter) and 3 mm thick acrylic cover. To insert, ticks were anesthetized briefly with 4 degree cold (wild ticks) or CO_2_ (all other experiments) and placed into assembled inserts with a 1 cm disc of 1.5% agar for humidity. In all recordings, temperature was 25 °C and relative humidity was kept above 80%.

### Ticks

#### Wild ticks

Wild ticks (Used in Fig. 1D-I) were collected by dragging through multiple independent drags between May 18th and June 23rd of 2022. Drags took place at Monkey Run Natural Area (Ithaca, NY, USA, 42° 27′ 47.4″ N 76° 25′ 37.8″ W) on dry days between 8 and 11AM (Sunrise/sunset varied from 6:18AM/8:24PM start to 5:29AM/8:47PM end). Each tick was pulled from drag and sexed on site, isolated with other same sex adults collected and brought to an incubator set and confirmed to be on a 12:12 LD cycle (Real time 7AM-7PM, light source 200 lx) and confirmed to be at 25 °C and 80% relative humidity via independent monitoring. Within the incubator ticks were combined with same-sex conspecifics in a plastic vial with a wet kimwipe for two days to acclimate to lighting conditions.

#### Lab-reared ticks

Lab-reared ticks (Used in Figs. 2, 3 and 4, S1–4) in were ordered from Biodefense and Emerging infectious Diseases Research Resources Repository (BEI Resources) and delivered from the CDC medical entomology lab in two separate shipments of 50 males and 50 females. After delivery, ticks were isolated by sex and kept in plastic vials at 22.5 °C 12:12LD 70–90% relative humidity with a wet kimwipe for 2 weeks. Once ticks were considered entrained and recordings began, ticks were kept at 25 °C either in the recording chamber or in a separate incubator in a vial with a wet kimwipe at 80 + % humidity and 25 °C.

### Behavior

#### Wild ticks circadian recording

At 7AM morning of the second day, males and females of each group (group size 5–7 for males, 8–11 for females) were placed into LOOC and recorded for 48 h. First 24 h were considered acclimation time and not scored. Second 24-h window was processed with T-Rex tracking software. For anaylsis, average speed among all tested ticks for every minute was compared during the 240 total minutes in each section of day and night for visualization purposes, or the 720 min for the entire day or night for statistical comparison. After recording finished, ticks were frozen and given to the Harrington Lab (Cornell University) for confirmation of sex and species. This was repeated for 3 independent recordings for each sex, and scored. Presence of pathogens was not checked, and is unknown. No ticks died during the ~ 48 h they were held and recorded, until they were frozen and discarded in ethanol after a single recording.

#### Lab-reared ticks circadian recording

As before, male or female groups of same-sex conspecifics (group size 22–24 for males, 22–25 females) were inserted into LOOC at lights on, recorded in an incubator confirmed to be on a 12:12 LD cycle and confirmed to be at 25 °C and 80% relative humidity via independent monitoring. Recording lasted for 72 h, first 24 h were considered acclimation time and not used, final 48 h were used for tracking. This was repeated for 3 independent recordings over both batches for each sex. Following the conclusion of circadian experiments, the lab-reared ticks were used for subsequent assessments of activity and sleep. Experiments were performed in two batches over 6 months following receipt of two separate shipments of 50 males and 50 females. Over the 1-year period, from 200, two male ticks escaped, and one male and four females died, presumably by drowning in their temporary housing vials. After 6 months of use of both batches, ticks were frozen and discarded in ethanol.

#### Light cycle-independent activity assessments

Both male and female CDC Colony ticks were entrained at 12:12 LD for four weeks before experiments began. Either at roughly ZT5 (Extended day) or exactly ZT0 (Extended night), males and females were CO_2_ anesthetized and placed in LOOC for 80 h. During the following day or night, respectively, the power source for the incubator lights was either moved to an outlet with constant power or unplugged. For extended day, analysis of videos started at ZT6 which is minute 0 on the graph.

#### Homeostatic activity assessment

Female CDC Colony ticks entrained to 12:12 L:D for months were CO_2_ anesthetized at ZT6, placed into LOOC and recorded for 48 h. After the first night, light control was reset to turn off after 6, 12, or 18 h. Activity of ticks were recorded for the 6 h before and 6 h after lights off. The integral of each activity curve was taken and compared for statistical analysis.

#### Stimulation/reversible quiescence assessment

All stimulations were performed on CDC Colony ticks between ZT4 and ZT7 on consecutive days. Groups of 25 female ticks were acclimated in LOOC for 10 min, recorded for 21 min with stimulus provided on the 10th minute, then left in LOOC, undisturbed for 20 min before the next trial. For mock stimulus, insert with arena was removed and top slid to expose circle hole, air puffer was pushed up to hole but no air was ejected. Vibration was applied by removing the insert and placing it on a vortexer (JT-14 brand) for 5 s before returning it to the chassis. Audio for sound sweep was played at 100 decibels from a bluetooth speaker 30 cm from LOOC. Sound sweep was a progression of sound from 20 Hz to 20 kHz over 30 s. For air puffs, a handheld dust blower (JJC soft tip silicone air blower) was preincubated in incubators at 4 °C, ambient temperature (25 °C), or warm (37 °C) for at least 30 min before air was ejected from dust blower into the LOOC insert. For CO_2_, the blower was manually filled with pure, room-temperature CO_2_ by a CO_2_ gun (Genesee Scientific) and ejected in the same manner as air puffs. Order of trials was randomized for roughly 5 stimuli per day, with the exception of CO_2_ stimulus, which was always performed last as ticks remained aroused for a prolonged period after CO_2_ puffs. Activity was scored both with tracking software and manually, single blind, by an undergraduate.

#### Day/night arousability assessment

25 °C air puffs were delivered to CDC Colony ticks at ZT4 (4 h after lights off) and ZT20 (4 h before lights off) in randomized order with six different groups of male or female ticks. After a ZT0 start time, puffs were delivered on the second night of recording and either the second or third day to prevent order bias. Recording was clipped to include only the 37 min before and after each stimulus.

### Tracking software

#### Long-term recording

LOOC output videos are converted to MP4 by VideotoVideo (https://www.videotovideo.org/). Videos are cut starting either at ZT0 of the first morning after video start (Fig. 1D–E, 2A–B) or 1 h after video start (Fig. 2G–H, S1A–B) into consecutive 4-h chunks until recording terminated and converted to grayscale with ffmpeg (https://ffmpeg.org/). Video files are then put through the T-Rex software workflow, starting with acquisition via Tgrabs (baseline settings: tgrabs -i input.mp4 -blob_size_range [0.5,2]). Reference image produced by Tgrabs is manually edited in MS Paint to remove dark pixels from ticks and create a homogenous reference background. T-Rex is run on the PV file and reference image output by Tgrabs (baseline settings: trex -i input [0.5,2] -blob_split_global_shrink_limit 0.1 -blob_split_max_shrink 0.1 -track_max_individuals 0 -meta_real_width 6.4 -match_mode automatic)(https://trex.run/docs/). In T-Rex, fidelity of tracking is manually assessed, if tracking errors are discovered, video is further cut into shorter chunks and/or Tgrabs input settings are adjusted. When the video has no tracking errors, arena diameter is set to 6.4 cm to calibrate pixel-length measurements and NPZ files are saved. Data is processed in Anaconda Jupyter Notebook (https://jupyter.org/) with 1 min binning and each 4-h chunk is plotted in series with Graphpad Prism software (https://www.graphpad.com/), adding 0’s for any tick that did not move throughout the chunk and thus not tracked.

#### Stimuli

LOOC output videos are converted to MP4 by VideotoVideo (https://www.videotovideo.org/). Videos are cut 1 min after start until the last stable frame before stimulus (~ 11 min). The video is cut a second time starting at the first frame with a stable background after stimulus until recording terminated. Videos are converted to grayscale with ffmpeg (https://ffmpeg.org/). Video files are then put through the T-Rex software workflow, starting with acquisition via Tgrabs (baseline settings: tgrabs -i input.mp4 -blob_size_range [0.5,2]). Reference image produced by Tgrabs is manually edited in MS Paint to remove dark pixels from ticks and create a homogenous reference background. T-Rex is run on the PV file and reference image output by Tgrabs (baseline settings: trex -i input [0.5,2] -blob_split_global_shrink_limit 0.1 -blob_split_max_shrink 0.1 -track_max_individuals 0 -meta_real_width 6.4 -match_mode automatic)(https://trex.run/docs/). In T-Rex, fidelity of tracking is manually assessed, if tracking errors are discovered, video is further cut into shorter chunks and/or Tgrabs input settings are adjusted. When the video has no tracking errors, arena diameter is set to 6.4 cm to calibrate pixel-length measurements and NPZ files are saved. Data is processed in Anaconda Jupyter Notebook (https://jupyter.org/) with 1 min binning and each ~ 10-min chunk is plotted with partner in Graphpad Prism (https://www.graphpad.com/), adding 0’s for any tick that did not move throughout the chunk and thus not tracked.

### Manual activity assessment

#### Long-term recording

For daytime activity, the same videos used for tracking were scored manually, noting time moving and time spent inactive-including either in sleep pose or time where limbs were not necessarily in sleep pose but torso position did not change. This data was used to calculate total activity time for each tick and to complement group population averages shown in activity data.

#### Response to stimuli

Videos of time before and after indicated stimulus was provided were scored single-blind by an undergraduate. For each minute, each tick was assigned an activity score; 1 if no activity was observed, 2 if prothoracic leg waving was observed but the animal did not move, and 3 if any walking was observed during the one-minute window. All recorded activity for all ticks/trials n = 22–25, 4–6 trials, was plotted.

#### Respirometry

Prior to respirometry recordings, CDC Colony ticks were held for multiple days at 22 °C and 12:12 LD photoperiod. At approximately ZT-0 on the day of testing, ticks were transferred (one tick per chamber per day) to a 1-mL respirometry chamber in a 22 °C incubator. Dry CO_2_-free air was pumped through an Alicat mass-flow controller at 19 mL/min. To minimize dehydration stress, post-controller airstream was passed through a Naflon humidifier RH ~ 90%. CO_2_ release was measured using a Li-Cor 6262 infrared detector. To minimize gas mixing problems in the respirometry chamber and downstream, a minimum length of narrow-gauge low-permeability PharMed tubing connected the chamber to the Li-Cor detector.

Respirometry data were collected for a 24-h period and analyzed using Expedata 1.9.17 software (Sable Systems International, Las Vegas, Nevada USA). During the majority of each recording, CO_2_ was released intermittently in bursts of ~ 1–3 nL. Burst volumes were calculated using Expedata's peak analysis routine. Manual integration was performed when Expedata could not resolve peaks. Preliminary experiments indicated that inter-burst CO_2_ levels were indistinguishable from an empty chamber, so inter-burst CO_2_ release was assumed to be zero. Inter-burst portions of the recordings were therefore used for baseline correction using Expedata.

Average metabolic rates during discontinuous CO_2_ release were calculated by integrating multiple consecutive peaks (minimum of 3 peaks, typically 6–10). Burst frequencies were measured by measuring the length of time between the onset of consecutive bursts. When ticks were not engaged in discontinuous CO_2_ release, metabolic rates were calculated from the average CO_2_ release above adjacent inter-burst (baseline) periods. Ticks were frozen at − 20 °C after respirometry and weighed to a precision of 1 µg using a microbalance C-30 (CAHN).

### Supplementary Information

Supplementary Figure S1.

Supplementary Figure S2.

Supplementary Figure S3.

Supplementary Figure S4.

Supplementary Movie S1.

Supplementary Movie S2.

Supplementary Legends.

## Data Availability

Lead contact: Further information and requests for resources and reagents should be directed to and will be fulfilled by the lead contact, Matthew R. Meiselman (matthew.meiselman@unlv.edu).

## Data availability

This study did not generate new unique reagents. All reagents used in the study are available upon request. All data including videos used for this study are available upon request to lead contact, Matthew R. Meiselman (matthew.meiselman@unlv.edu).
