# Supplementary figures and images for: Circadian regulation of locomotion, respiration, and arousability in adult blacklegged ticks (Ixodes scapularis)
Source: Sci Rep. 2024 Jun 26;14:14804. doi: 10.1038/s41598-024-65498-z (PMC11208436; doi:10.1038/s41598-024-65498-z)

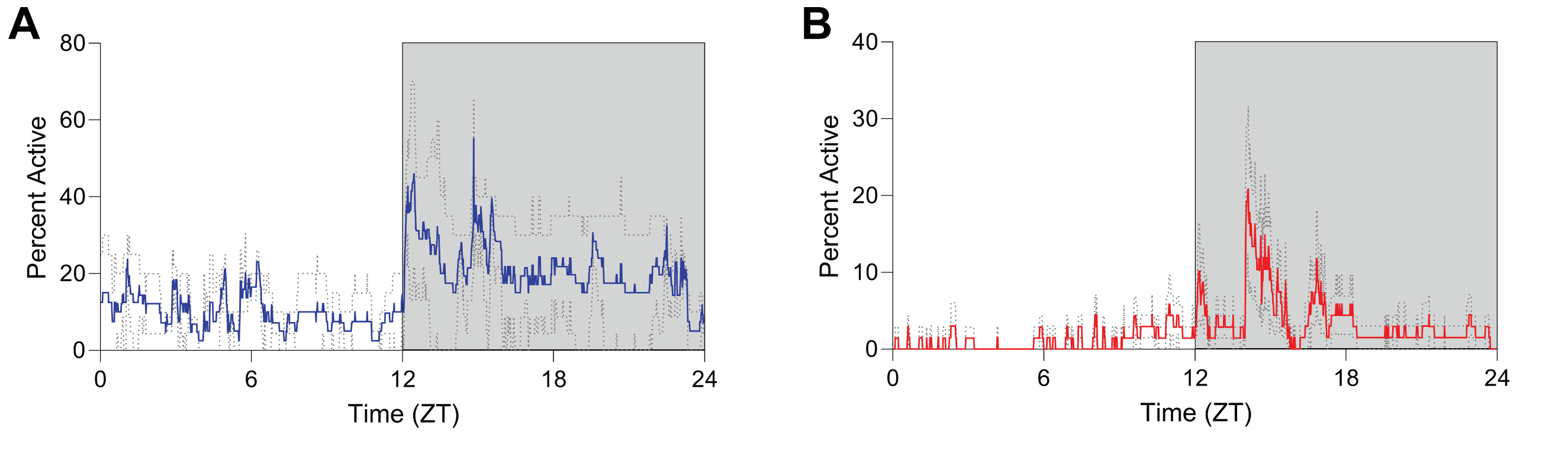

Supplement: Supplementary file 1 — Supplementary Figure S1. [file 41598_2024_65498_MOESM1_ESM.tif]

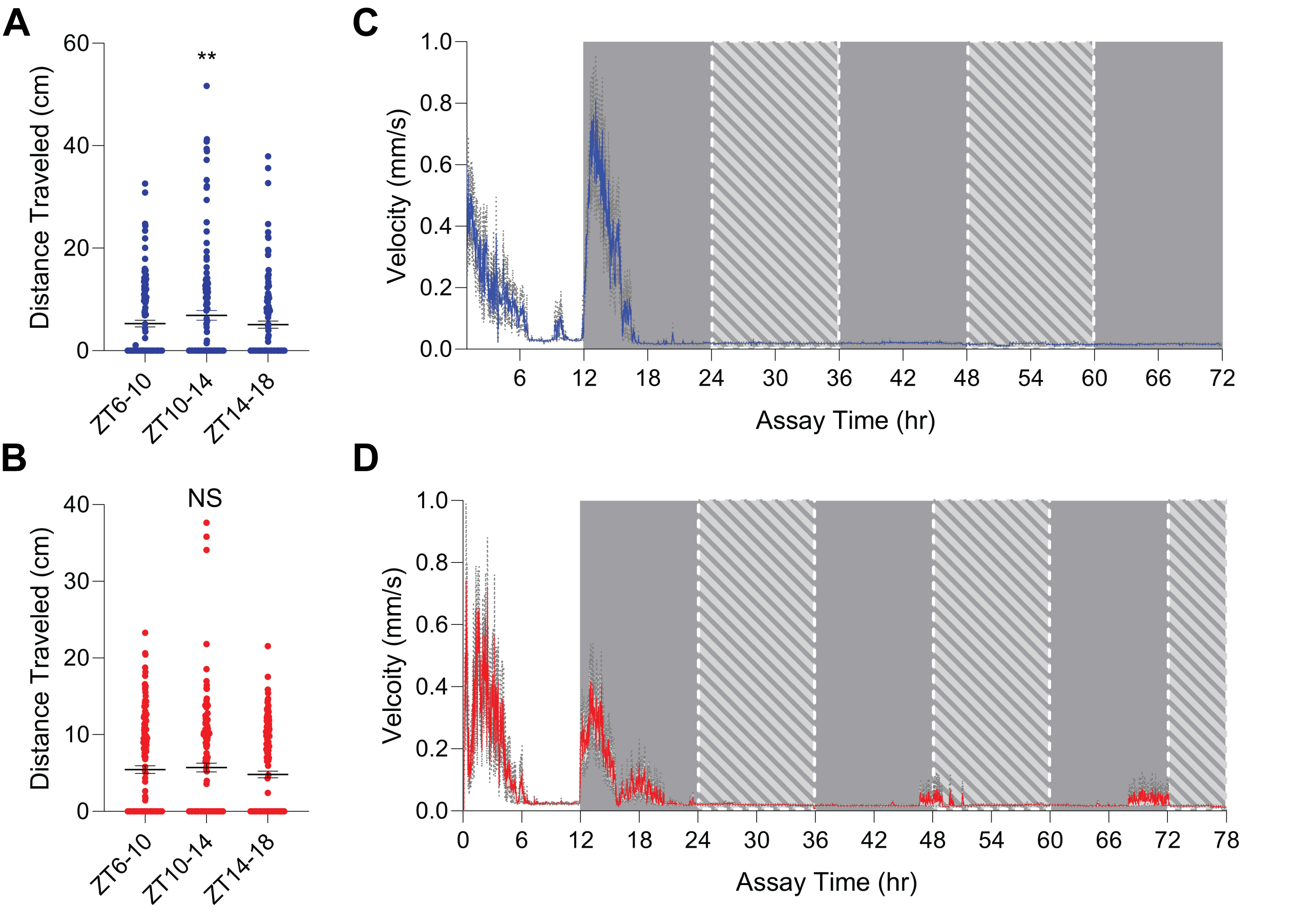

Supplement: Supplementary file 2 — Supplementary Figure S2. [file 41598_2024_65498_MOESM2_ESM.tif]

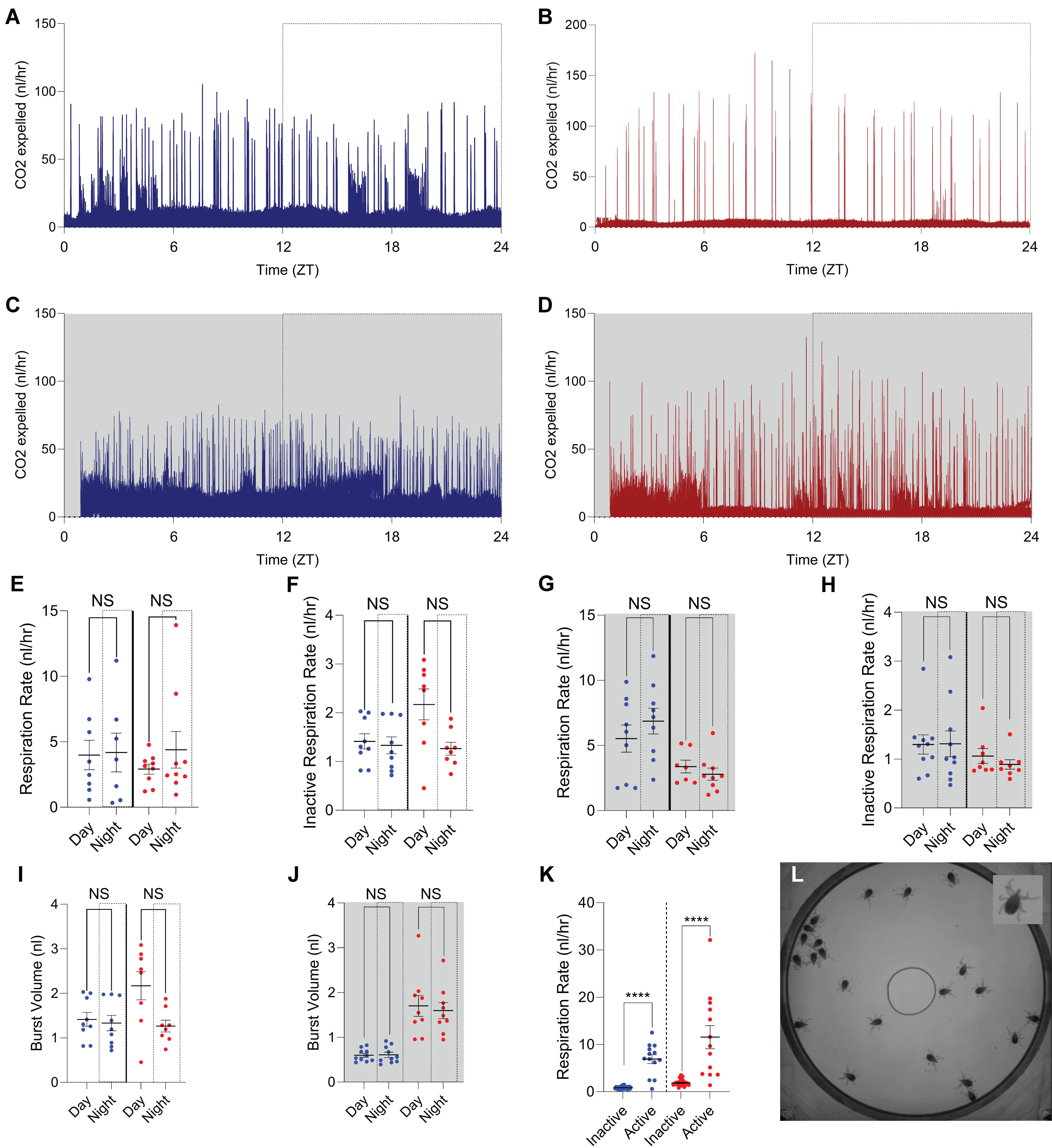

Supplement: Supplementary file 3 — Supplementary Figure S3. [file 41598_2024_65498_MOESM3_ESM.tif]

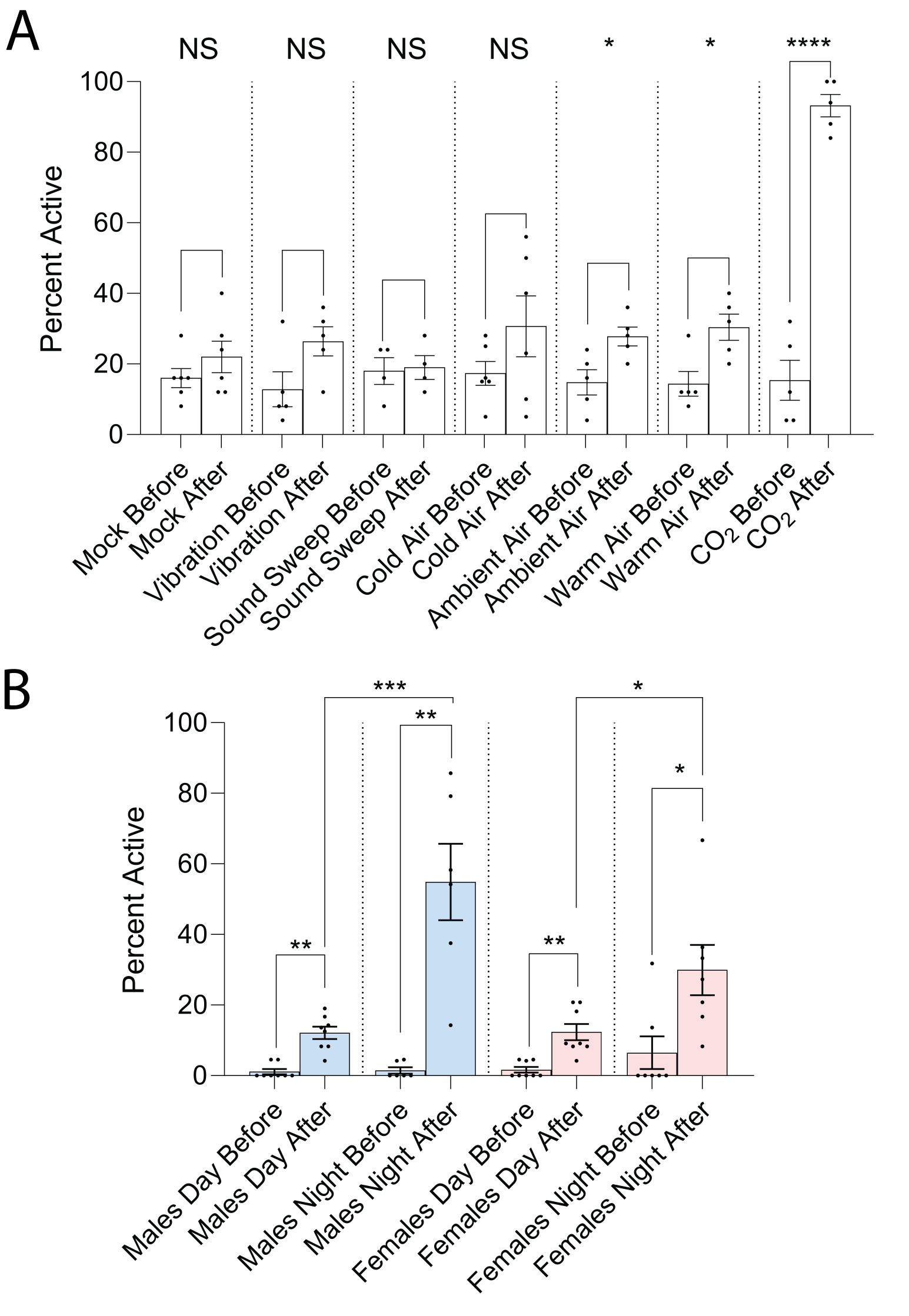

Supplement: Supplementary file 4 — Supplementary Figure S4. [file 41598_2024_65498_MOESM4_ESM.tif]
