# Supplementary material for: Circadian regulation of locomotion, respiration, and arousability in adult blacklegged ticks (Ixodes scapularis)
Source: Sci Rep. 2024 Jun 26;14:14804. doi: 10.1038/s41598-024-65498-z (PMC11208436; doi:10.1038/s41598-024-65498-z)
Supplement: Supplementary file 7 — Supplementary Legends. [file 41598_2024_65498_MOESM7_ESM.docx]

**SUPPLEMENTAL FIGURES AND LEGENDS**

**S1 Fig.** **(A-B)** Manually scored activity data for males **(A)** and females **(B)**, showing percent of all ticks active within a group (22-25 per group, 3 groups each), gray box indicated circadian night, dotted lines indicate S.E.M.

**S2 Fig.** **(A-B)** Total distance traveled per tick during the first day of the constant light regime at ZT 6-10, ZT10-14 (expected dark), and ZT14-18 for males **(A)** and females **(B)**. Error bars represent SEM, Friedman’s pairwise test, NS, p>0.05, *p<0.05.

**(C-D)(C)** Average velocity per tick for male ticks (n=66) exposed to constant dark after first night for 2.5 days (3600 minutes). Gray indicates dark onset, white dotted lines indicate expected day where light would have turned on if cycling continued, light grey dotted line is SEM. **(D)** Average velocity per tick for female ticks (n=71) exposed to constant dark after first night for 2.63 days (3840 minutes). Gray indicates dark onset, white dotted lines indicate expected day where light would have turned on if cycling continued, light grey dotted line is SEM.

**S3 Fig.** **(A,B)** Example traces of CO_2_ concentration in flow-through for 9 male **(A)** and 8 female **(B)** ticks during a day in a constant light regime (LL), overlaid. Dotted box indicates expected night (ZT12-24).

**(C,D)** Example traces of CO_2_ concentration in flow-through for 9 male **(C)** and 9 female **(D)** ticks during a day in a constant dark regime (DD), overlaid. Dotted box indicates expected night (ZT12-24).

**(E)** Total CO_2_ respiration rate for 8 male (left, blue) and 9 female (right, red) ticks represented in A and B comparing circadian day (white) to circadian night (dotted box) by sex, in constant light conditions (LL). Student’s t-test, error bars indicate mean +/- SEM. NS, p>.05.

**(F)** CO_2_ respiration rate comparing only periods of inactivity for each male (left, blue) and female (right, red) represented in LL regime (A,B) comparing circadian day (white) and expected night (dotted lines)(Students t-test, error bars indicate mean +/- SEM. NS, p>.05).

**(G)** Total CO_2_ respiration rate for 9 male (left, blue) and 9 female (right, red) ticks represented in A and B comparing expected day (gray) to circadian night (dotted box) by sex, in constant dark conditions (DD). Student’s t-test, error bars indicate mean +/- SEM. NS, p>.05.

**(H)** CO_2_ respiration rate comparing only periods of inactivity for each male (left, blue) and female (right, red) represented in DD regime (A,B) comparing expected day (gray) and night (dotted box)(Students t-test, error bars indicate mean +/- SEM. NS, p>.05).

**(I)** Average volume of CO2 released during bouts of discontinuous gas exchange for males (left, blue) and females (right, red) in the LL regime (A,B) during day (white boxes) and expected night (dotted box)(n=8-9, pairwise t-test, NS, p>0.05).

**(J)** Average volume of CO2 released during bouts of discontinuous gas exchange for males (left, blue) and females (right, red) in the DD regime (C,D) during expected day (gray) and expected night (dotted box)(n=9, pairwise t-test, NS, p>0.05).

**(K)** Comparison of tick respiration during stationary periods (inactive) and during activity bouts (active) compared for males (left, blue) and females (right, red). n=13 per sex, student’s t-test, error bars indicate mean +/- SEM. ****p<0.001.

**(L)** Example frame ~5 minutes after night where ticks are not moving, but arms are extended. Closeup of an individual tick in the top right inset.

**S4 Fig.** **(A)** Percent of ticks in a given group moving in the entire 10-minute window before and after each stimulus. N=20-25 ticks per group, 4-6 groups per stimulus (Students t-test, error bars indicate mean +/- SEM. NS, p>.05, *,p<0.05, ****, p<0.0001).

**(B)** Percent of ticks in a given group moving in the entire 37-minute window before and after air puff stimulus. N=21-24 ticks per group, 6-8 groups per stimulus (Paired t-test for comparisons of active ticks before and after stimuli, unpaired t-test comparing percentage active after day stimulus to after night stimulus, error bars indicate mean +/- SEM. *,p<0.05,**,p<0.01,***, p<0.001).

**S1 Movie Male** **Arousal behavior**.

Close up video of a male tick roused from diurnal sleep by breath at 5 seconds.

**S2 Movie Female** **Arousal behavior**.

Close up video of a female tick roused from diurnal sleep by breath at 4 seconds.
